# Genomic Analysis Using Bayesian Methods under Different Genotyping Platforms in Korean Duroc Pigs

**DOI:** 10.3390/ani10050752

**Published:** 2020-04-25

**Authors:** Jungjae Lee, Yongmin Kim, Eunseok Cho, Kyuho Cho, Soojin Sa, Youngsin Kim, Jungwoo Choi, Jinsoo Kim, Junki Hong, Taejeong Choi

**Affiliations:** 1 Jung P & C Institute, Inc., 1504 U-TOWER, Yongin-si, Gyeonggi-do 16950, Korea; 2 National Institute of Animal Science, Rural Development Administration, Cheonan 331-801, Korea; 3 College of Animal Life Sciences, Kangwon National University, Chuncheon 24341, Korea

**Keywords:** genome-wide association study, genomic breeding value, Duroc, single nucleotide polymorphism

## Abstract

**Simple Summary:**

This study investigated the informative regions and the efficiency of genomic predictions for backfat thickness, days to 90 kg body weight, loin muscle area, and lean percentage in Korean Duroc pigs. The several regions of the genome were identified and a significant marker was found near the MC4R gene for growth and production-related traits. No differences in genomic accuracy were identified on the basis of the Bayesian approaches in these four growth and production-related traits. The genomic accuracy is improved by using deregressed estimated breeding values including parental information as a response variable in Korean Duroc pigs.

**Abstract:**

Genomic evaluation has been widely applied to several species using commercial single nucleotide polymorphism (SNP) genotyping platforms. This study investigated the informative genomic regions and the efficiency of genomic prediction by using two Bayesian approaches (BayesB and BayesC) under two moderate-density SNP genotyping panels in Korean Duroc pigs. Growth and production records of 1026 individuals were genotyped using two medium-density, SNP genotyping platforms: Illumina60K and GeneSeek80K. These platforms consisted of 61,565 and 68,528 SNP markers, respectively. The deregressed estimated breeding values (DEBVs) derived from estimated breeding values (EBVs) and their reliabilities were taken as response variables. Two Bayesian approaches were implemented to perform the genome-wide association study (GWAS) and genomic prediction. Multiple significant regions for days to 90 kg (DAYS), lean muscle area (LMA), and lean percent (PCL) were detected. The most significant SNP marker, located near the MC4R gene, was detected using GeneSeek80K. Accuracy of genomic predictions was higher using the GeneSeek80K SNP panel for DAYS (Δ2%) and LMA (Δ2–3%) with two response variables, with no gains in accuracy by the Bayesian approaches in four growth and production-related traits. Genomic prediction is best derived from DEBVs including parental information as a response variable between two DEBVs regardless of the genotyping platform and the Bayesian method for genomic prediction accuracy in Korean Duroc pig breeding.

## 1. Introduction

Genomic selection (GS) has been widely applied to several species, for example, pigs, chickens, beef, and dairy cattle, using commercial single nucleotide polymorphism (SNP) genotyping platforms from Illumina, GeneSeek-Neogen, and Affymetrix. These arrays estimate genomic-enhanced estimated breeding values (GE-EBVs), which are blended with classical estimated breeding values (EBVs) from classical genomic best linear unbiased prediction (BLUP) and molecular breeding values (MBVs) from summation of single nucleotide polymorphism (SNP) marker effects for genotyped animals. The most important parameter in genomic prediction modeling is the accuracy of genomic prediction for the estimation of GE-EBVs because the weights are determined on the basis of that parameter when blended with traditional EBVs and MBVs in a “correlated traits” approach [1]. Two terms in the classical concept of quantitative genetics in the formula of genetic progress ($\Delta G=\frac{i\times r\times \sigma_{g}}{L}$) that are directly affected by the implementation of genomic selection in the pig industry are the generation interval (L) and accuracy (r).

Genetic improvements are achieved by reducing the generation interval and increasing the accuracy through genomic selection modeling in dairy and beef cattle. However, in pigs, genetic improvements with the generational interval parameter are limited by rapid generational turnover. Therefore, increased accuracy of genetic predictions may be the largest parameter impacting genomic selection in pig breeding [2]. These authors [2] also reviewed the accuracy of genomic prediction for maternal, performance, and carcass traits in pigs. Breeders of various animal species have conducted research using various prediction models with the aim of increasing the accuracy of genomic prediction for more reliable GE-EBVs. However, these models are affected by several factors, such as the size of the reference data, which mean having both genomic and phenotypic data [3,4], density of genotyping platforms [5,6,7,8], relationships between training and testing sets in the process of cross validation for genomic accuracy [9], and choice of response variables in genomic prediction models [8]. In addition, the models were also affected by the choice of covariates (individual SNP vs. haplotype) in genomic prediction modeling [10], choice of penalty or a priori density in statistical methods (e.g., regression on SNP marker) [11], and causative variants or SNPs in strong linkage disequilibrium (LD) with causative variants [12].

The objectives of this study were to (1) identify informative genomic regions through a genome-wide association study (GWAS) and (2) to investigate and compare the accuracy of genomic prediction of two genomic evaluation methods using two Bayesian methods (BayesB and BayesC) under two medium-density, SNP genotyping platforms with two response variables (DEBVexcPA and DEBVincPA). This is the first study to assess the accuracy of genomic prediction for growth and production-related traits in Korean Duroc pig populations.

## 2. Materials and Methods

### 2.1. Genotype and Phenotype Data Editing and Imputation

A total of 1026 Duroc pigs were genotyped. These animals were genotyped with two medium-density SNP genotyping platforms, including 487 genotyped by the Illumina PorcineSNP60 version 2 (Illumina, Inc., San Diego, CA, USA) and 539 genotyped by GeneSeek-Neogen PorcineSNP80 (BeadChip Neogen Agrigenomics, Lincoln, NE, USA), respectively. They consisted of 61,565 and 68,528 SNP markers, respectively. The quality control measures in SNP markers and animals were performed by excluding 7849 and 7758 unmapped SNPs, 1458 and 3273 SNPs on sex chromosomes, 6399 and 1613 SNPs with a poor call rate (<0.90), 17 and 18 SNPs with a poor call rate for a duplicate SNP map-position for SNP markers, 7 and 5 animals with poor call rates (<0.90), 8 and 34 animals that did not match with phenotypes for each Illumina PorcineSNP60 version 2 and GeneSeek-Neogen PorcineSNP80 genotyping platform, and 3 animals with a poor call rate for duplicate genotypes between two SNP genotyping platforms. Consequently, the number of available SNP markers was 45,840 and 55,866 for 60K and 80K SNP panels, respectively, leaving 472 and 500 animals for 60K and 80K SNP panels, respectively, for use in further genome-wide association studies, and genomic prediction modeling.

The imputation processes of these two medium density SNP panels (Illumina60K and GeneSeek80K) were separately performed using the following two steps: (1) FImpute version 2.2 [13] imputed missing SNP genotypes of two SNP panels (50K and 80K) on the basis of the information for each marker map to be used for the reference panel and (2) FImpute version 2.2 [13] imputed between two SNP panels, from Illumina60K to GeneSeek80K and from GeneSeek80K to Illumina60K. Finally, we accepted two kinds of reference population for further genomic analysis, consisting of 972 animals in the imputed 60K and 80K data because there were no duplicate genotypes between the Illumina60K and GeneSeek80K.

### 2.2. Deregression of Expected Breeding Values (DEBVs) for Response Variables

A multitrait animal model with 46,305 phenotypic data recorded from 2005 to 2017 and 72,781 pedigree records was applied to estimate the variance components and genetic parameters (Table 1).

This was required as a priori information for the genomic prediction model and for EBVs and corresponding reliabilities for the genotyped animals and their sires and dams. These analyses used the ASReml version 4.1 software [14] for four growth and production-related traits: backfat thickness (BFAT), days to 90 kg body weight (DAYS), loin muscle area (LMA), and lean percent (PCL). Phenotypes were adjusted for fixed effects using contemporary groups comprising of farm, birth-year, season, and sex effects. A common litter environment effect was also included in a multitrait animal model for those parameters and EBV. We used the methodology provided by Garrick et al. [15] for the two kinds of DEBVs, which were (1) a combination of deregression (dividing by the reliability of the EBV) and adjustment for ancestral information (i.e., parental average, which only contained their own and the descendant’s information, hereafter called “DEBVexcPA”), and (2) in contrast to Garrick et al. [15], the parent average EBV (PA) was added back to the DEBV (hereafter called “DEBVincPA”) to account for breed and family differences in subsequent analyses. These two DEBVs (DEBVexcPA and DEBVincPA) were obtained using Equation (1):

(1) $$DEBV_{i}=\left(PA\right)+\frac{{\hat{g}}_{i}-PA}{r_{i}^{2}},$$

with the corresponding weighting factors using Equation (2):

(2) $$w_{i}=\frac{\left(1-h^{2}\right)}{\left\{c+\left[\left(1-r_{i}^{2}\right)/r_{i}^{2}\right]\right\}h^{2}},$$

where ${\hat{g}}_{i}$ is the EBV (estimated breeding value) of the individual, PA is its parent average, *h*^2^ is the heritability, $r_{i}^{2}$ is the reliability of the EBV of the individual, and c is the proportion of genetic variation that could not be explained by the markers. In this study, c was assumed to be equal to 0.40 and is the proportion of the genetic variance not explained by SNP markers, as suggested by Saatchi et al. [16]. After removing animals with a reliability of less than 0.10, 964 registered Duroc pigs remained for further analysis.

### 2.3. Statistical Method for Estimating SNP Effects

Two methods (BayesB [3] and BayesC [17], with π set to 0.99 and weighting factors), were used to estimate SNP marker effects using the GenSel4R software [18] for GWAS and genomic prediction models. The BayesB and BayesC methods use the mixture model that assumes some fraction π of SNP markers have zero effects and assumes that SNP markers have non-zero effects. The BayesB method uses the t-distribution a priori for the SNP marker effects and has locus-specific variances whereas the BayesC method uses the normal distribution a priori for the SNP marker effect and has a common variance [19]. For each trait, the model was fitted to estimate SNP marker effects for these two methods using Equation (3):

(3) $$y_{i}=\mu +\sum_{j=1}^{k}Z_{ij}u_{j}\delta_{j}+e_{i},$$

where $y_{i}$ is response variable (DEBVexcPA or DEBVincPA) on animal i for the respective trait, I? is the population mean, k is the number of markers, $Z_{ij}$ is allelic state at locus j in individual i, and $u_{j}$ is the random substitution effect for marker j, which follows a mixture distribution for this random substitution effect according to indicator variable ($\delta_{j}$). A random absent (0) or present (1) variable indicates the absence or presence of marker j in the model, with $u_{j}$ assumed normally distributed N(0, $\sigma_{u}^{2}$) when $\delta_{j}=1$, and $e_{i}$ is a random residual effect assumed normally distributed N(0, $\sigma_{e}^{2}$). The posterior distributions of the parameters and effects were obtained using Gibbs sampling, for a total number of 110,000 Markov chain Monte Carlo (MCMC) iterations, the first 10,000 of which were discarded for burn-in, before estimating posterior means of marker effects and variances, and a sampling interval (thinning) of 10. All procedures were implemented in GenSel4R software [18]. The convergence of MCMC iterations was tested by comparing results from three iteration lengths (75,000 vs. 110,000 vs. 150,000) with the first 10,000 cycles being discarded and having a sampling interval of 10. The differences in the posterior means of genetic and residual variances were negligible among three MCMC iteration lengths for all growth and productive-related traits (results not shown).

### 2.4. Identification of Significant Window Regions and SNP Markers

The 0.8% of additive genetic variance, which was estimated as a fraction of the total genetic variance explained by all SNPs, was used for the significance level of the putative informative 1 Mb window region. A total of 2454 1-Mb window regions located on autosomes were considered for two SNP genotyping platforms (Illumina60K and GeneSeek80K) in this analysis. The theoretical rate of the genetic variance could be assumed approximately 0.04% (100% /2454), but the stringent threshold of 0.8%, which is twenty times higher than the theoretical proportion was considered as the small reference set in Korean Duroc pigs. The Bayes factor (BF) was used to determine SNPs with a significant association within this region using Equation (4):

(4) $$BF=\frac{\hat{p_{i}}/(1-\hat{p_{i}})}{(1-\pi )/\pi },$$

where π is the prior probability and $\hat{p_{i}}$ is the posterior probability that an SNP was included in the model. Following the definitions of Kass and Raftery [20] for the strength of an association on the basis of the range of values, the SNP markers with a Bayes factor above 3.2 were considered as “suggestive evidence”, above 20 was described as “strong evidence” and above 100 was described as “decisive evidence”.

### 2.5. Accuracy of Genomic Prediction under a 10-Fold Cross-Validation

To account for the relatively small sample size of the prediction model, a 10-fold cross-validation strategy was used to estimate the accuracies of the genomic prediction models. Previous study related to the number of folds on the process of the cross validation have reported that trade-off effects were detected between the number of folds and the relationships between training and testing sets [8]. Nevertheless, we used a 10-fold cross-validation to maximize the size of the training data because of the limited reference data set in Korean Duroc pigs. For each trait of interest in this study (BFAT, DAYS, LMA, and PCL) and following the procedures outlined by Saatchi et al. [9], genotyped animals were split into ten groups using K-means clustering to reduce the relationships between training and testing populations. A total of 3821 elements of pedigree information related to the 964 genotyped Duroc pigs was used for K-means clustering, giving the number of individuals within each fold, and within and between fold averages of *a_max_* and *a_ij_*, and their standard deviations (Table 2).

Accuracies of genomic prediction were assessed by the correlation between the MBVs of genotyped animals from each validation set and their response variables, $r\left(\hat{y},\;y\right)$, where y is a vector of pseudo-phenotypes (DEBVexcPA or DEBVincPA) for the validation set and $\hat{y}$ is a vector of MBV for the corresponding animals in y.

## 3. Results and Discussion

### 3.1. Assessing the Accuracy of Imputation

**Figure 1:**
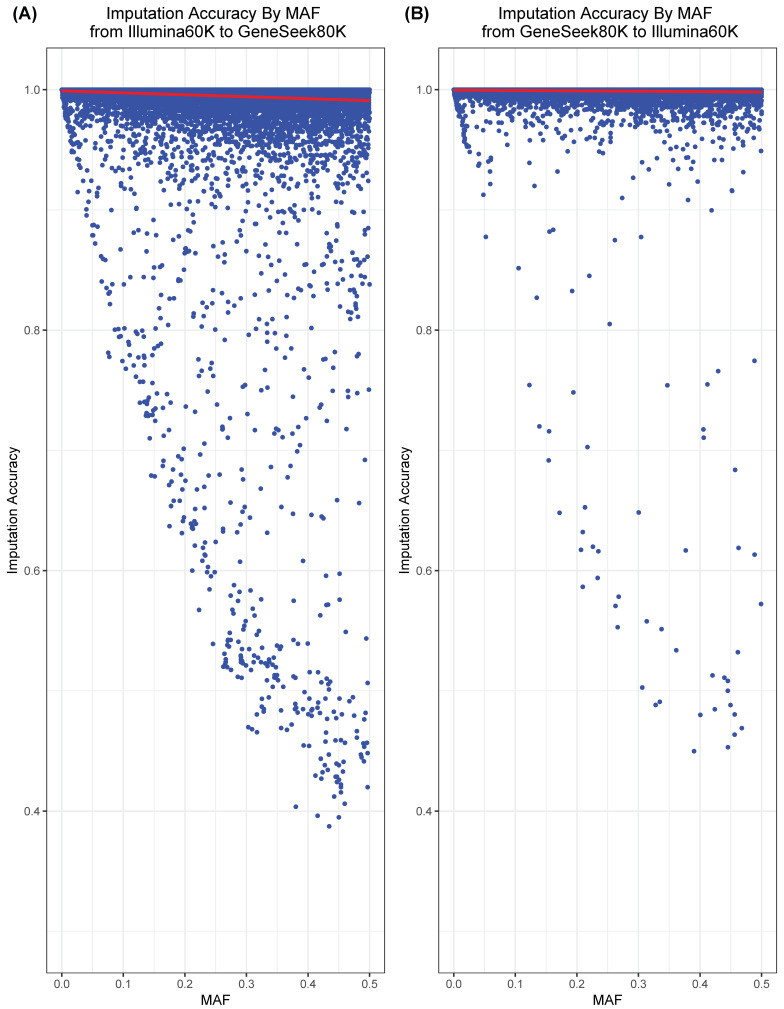
Imputation accuracy computed using the proportion of correctly imputed genotypes by minor allele frequency (MAF). Imputation accuracy computed (**A**) from the Illumina60K to GeneSeek80K and (**B**) from the GeneSeek80K to Illumina60K.

The imputation process was performed to test the imputation accuracy of two SNP genotyping platforms, the Illumina60K and GeneSeek80K. The accuracy of imputation with a higher minor allele frequency (MAF) was lower than for those with a lower MAF for both SNP genotyping platforms (Figure 1).

These results are consistent with the results of Badke et al. [21], who showed that the proportion of correctly imputed alleles decreased by increasing the number of SNPs with a high MAF in Yorkshire pigs. Using dairy cattle, Ma et al. [22] showed that the imputation accuracies were lower with a higher MAF across available imputation programs [13,23,24,25,26]. The accuracies of imputation using simulation studies were 98.6% from the Illumina60K to GeneSeek80K SNP panel and 99.4% from the GeneSeek80K to Illumina60K SNP panel. The accuracies of imputation were similar and consistent across chromosomes for imputation to both SNP platforms from the other SNP platform, likely because the proportion of common SNP markers between the two SNP genotyping platforms (Illumina60K: 59.1% and GeneSeek80K: 53.1%) was high.

### 3.2. Genome-Wide Association Study (GWAS) for Growth- and Production-Related Traits

**Figure 2:**
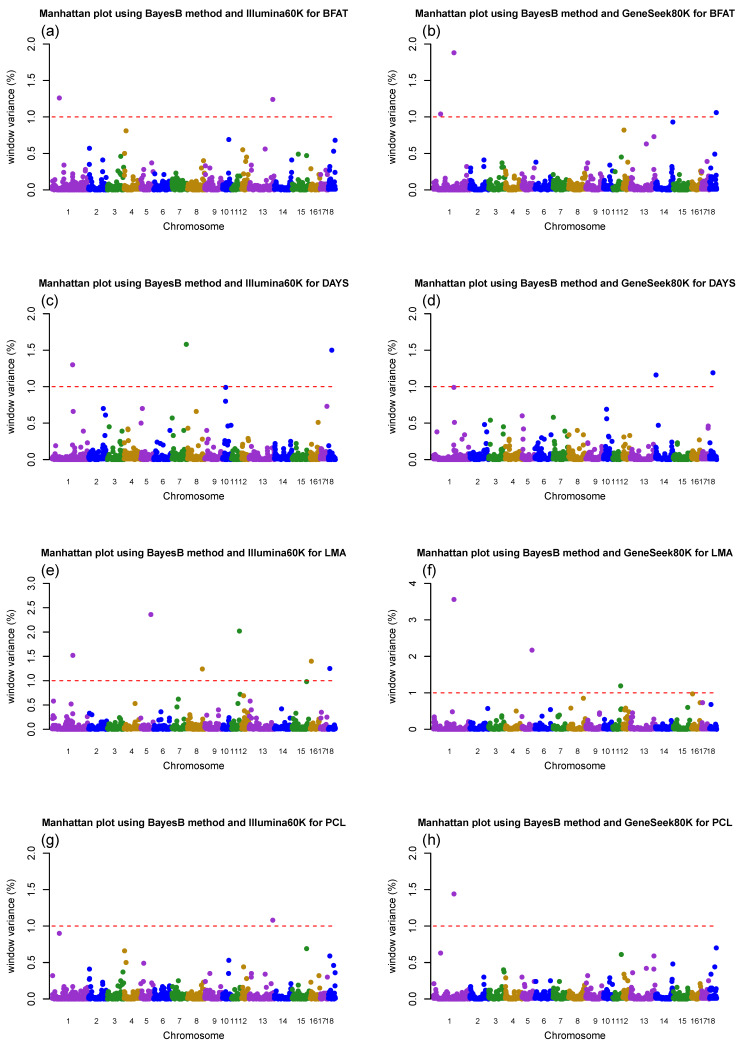
Manhattan plot of the GWAS result of 18 porcine autosomes using the BayesB method and two SNP genotyping platforms, the Illumina60K and GeneSeek80K. The y-axis indicates window variance (%), and the x-axis represents the pig autosomal chromosome physical map. The red dotted horizontal lines indicate that the threshold of the percent variance of the 1 Mb genomic region used was above 1.0% to identify associations with two SNP genotyping platforms and traits: (**a**) backfat thickness (BFAT) with the Illumina60K, (**b**) BFAT with the GeneSeek80K, (**c**) days to 90 kg body weight (DAYS) with the Illumina60K, (**d**) DAYS with the GeneSeek80K, (**e**) loin muscle area (LMA) with the Illumina60K, (**f**) LMA with the GeneSeek80K, (**g**) lean percent (PCL) with the Illumina60K, and (**h**) PCL with the GeneSeek80K.

GWAS for growth and production traits was performed using two commercially developed Porcine SNP genotyping platforms (Illumina60K and GeneSeek80K) to identify the most informative window regions and significant SNP markers based on the Bayes factor within these regions. GWAS analyses using BayesB with a high value of π (0.99) and a DEBVincPA response variable for growth and production-related traits in Duroc pigs were chosen because the informative window region and significant SNP markers were similarly distributed across the three response variables and Bayesian methods. The results of these associations are shown in Table 3 and Table 4, and Figure 2.

Three and four informative windows (1 Mb) were detected for BFAT using the Illumina60K panel and GeneSeek80K panel, respectively. The most significant window was identified on Sus scrofa chromosome (SSC)1 at 62 Mb using the Illumina60K panel and on SSC1 at 178 Mb using the GeneSeek80K panel, which explained 1.26% and 1.88% of genetic variance, respectively. Significant SNP markers, based on the Bayes factor, common to both two panels were ALGA0003581 and ALGA0003587, which were located on SSC1 at the 62 Mb position nearby the CGA gene. For DAYS, we detected five informative quantitative trait loci (QTL) using the Illumina60K panel and three informative QTLs using the GeneSeek80K panel. The regions of SSC7 at 124 Mb (1.58%) and SSC18 at 29 Mb (1.19%) were the most informative 1 Mb window regions in GWAS for DAYS using the Illumina60K and using GeneSeek80K, respectively. The common significant SNPs were ALGA0097693 (located on SSC18 at the 29 Mb position between TSPAN12 and CFTR) and ASGA0004988 (located on SSC1 at the 177 Mb position between RNF152 and MC4R) in both panels. We identified six significant regions using the Illumina60K panel and five significant regions using the GeneSeek80K panel for LMA. The most significant region using the Illumina60K was detected on SSC5 at 87 Mb (2.36%), and SSC1 at the 178Mb (3.56%) region was detected when using GeneSeek80K. Two informative QTLs were detected using the Illumina60K and GeneSeek80K panels for PCL. In addition, common significant SNPs were not identified using either panel. The GeneSeek80K genotyping panel contained more SNP markers in major genes than the Illumina60K genotyping panel (i.e., MC4R). As a result, the informative SNP markers not included in the Illumina60K panel were detected using the GeneSeek80K panel. Interestingly, the WU_10.2_1_178188861 SNP located by the GeneSeek80K panel was associated with all growth and production traits except DAYS, which is not included in the Illumina60K panel and is located on SSC1 at the 178 Mb position between *RNF152* and MC4R. For DAYS, ASGA0004988, which was positioned on SSC1 at 177 Mb, was detected as an informative SNP marker, but the nearest gene was MC4R. The MC4R gene is a major determinant of the nervous system and plays a substantial role in the regulation of food intake, energy balance, and body weight in mammals [27,28,29]. Previous studies [29,30,31,32] have reported the identified QTL near the MC4R gene located at 178 Mb on SSC1 as Sscrofa10.2. Our findings related to the 178 Mb region on SSC1, along with other significant regions, were consistent with previously identified regions that potentially impact growth and production traits in the Animal QTL database.

### 3.3. Accuracy of Genomic Prediction

#### 3.3.1. SNP Genotyping Platforms and Bayesian Methods

Table 2 shows that the data were successfully partitioned using the K-means clustering method for genomic evaluation, whereby the relatedness was maximized within each partitioned group and minimized between each partitioned group. The accuracy of the genomic prediction with BayesB using the Illumina60K ranged from 0.179 (BFAT) to 0.234 (LMA) and from 0.247 (BFAT) to 0.314 (LMA) for DEBVexcPA and DEBVincPA, respectively (Table 5).

A similar trend was observed with BayesC when using the GeneSeek80K, with a range of accuracies from 0.176 (BFAT) to 0.246 (LMA) and from 0.250 (BFAT) to 0.331 (LMA) for DEBVexcPA and DEBVincPA, respectively (Table 5). These results indicate similar levels of accuracy of genomic prediction regardless of the genotype platform or Bayesian method. However, a slight increase in the accuracy of genomic prediction was observed in DAYS (2%) with the DEBVexcPA response variable and LMA (2% and 3%) with the DEBVexcPA and DEBVincPA response variables when comparing the GeneSeek80K to the Illumina60K SNP genotyping platform. These comparisons between different SNP genotyping platforms have also been studied in beef and dairy cattle [7,33]. In cattle, the accuracies of genomic prediction were compared between moderate and high-density panels (50K and 777K) or between moderate-density genotype panels (50K and 80K). Pérez-Enciso et al. [7] observed that the reliabilities of genomic predictions did not increase when using a high-density SNP chip (HD) compared with a 50K SNP chip. Lee et al. [8] and Guo et al. [33] also reported no significant improvement in accuracy when using a 50K panel vs an 80K panel for Red Angus beef cattle in the United States. Overall, no significant improvements in prediction accuracies on the basis of SNP panel density have been observed from the results of previous genomic prediction studies (50K vs. 777K or 50K vs. 80K) because even though the number of SNPs increases, the panel may contain a small number of SNP markers in high LD with causative variants. In addition, simply increasing SNP markers instead of causal mutation may bring an additional source of noise to genomic prediction [5,7,8]. The results revealed a slight increase in genomic accuracy for DAYS and LMA with the BayesB method and the GeneSeek80K SNP platform compared with the Illumina60K SNP platform. This was because the GeneSeek80K SNP platform includes a causal variant in strong LD with the MC4R gene [2], which was the most informative for LMA (Table 4, Figure 2). These results are consistent with the results of Pérez-Enciso et al. [7] and Van et al. [12], suggesting that the inclusion of causative variants or SNPs with high LD with causative mutation improved the accuracy of genomic prediction.

#### 3.3.2. Response Variables (DEBVincPA and DEBVexcPA)

The average accuracies of genomic prediction across the growth- and production-related traits ranged from 0.177 (BFAT) to 0.244 (LMA) for the Illumina60K and GeneSeek80K when using DEBVexcPA as a response variable, and from 0.252 (BFAT) to 0.327 (LMA) for the Illumina60K and GeneSeek80K when using DEBVincPA as a response variable with 10-fold cross validation (Table 5). In the current study, we observed higher prediction accuracies when using DEBVincPA as a response variable compared with DEBVexcPA for all studied traits. Interestingly, the largest difference (+8.3%) in terms of average accuracies of genomic prediction between the two response variables was observed for the lowest heritable trait (LMA) when using DEBVincPA as a response variable. While DEBVexcPA [15] has the greatest numerical properties in addressing double counting by removing the parental contribution [8,33,34], our results showed a lower performance in prediction accuracies in comparisons of two response variables. Boddhireddy et al. [34] reported that using EBV without removing parental contributions as a response variable yielded greater prediction accuracies compared to using DEBVexcPA in both validation tests for US Angus beef cattle. Lee et al. [8], however, observed that the genomic accuracies obtained using DEBV after removing parental information as a response variable were higher than those obtained using DEBV without removing parental information in growth and carcass traits in US Red Angus beef cattle. The differences in genomic accuracies among the different panels were not significant for the traits used in this study. Although the GeneSeek80K panel contained a major marker, MC4R, this had no influence on genomic prediction; however, the genomic accuracy using this panel was approximately 3% higher than when using the Illumina60K panel in LMA.

An advantage of excluding the parent average (PA) was to avoid double counting. Otherwise using PA would shrink the individual EBV toward to the parent average [15]. However, the inclusion of PA after deregression added an advantage by accounting for the differences in PA among genotyped animals, such as between family differences [35]. For all studied traits, DEBVincPA, as the response variable, showed higher genomic accuracy than DEBVexcPA. This finding supports the result of Lee et al. [36] that DEBVincPA compared with other response variables (EBV and DEBVexcPA) was the most advantageous genomic prediction in Korean Yorkshire pigs. However, because this result is from a relatively small training size, we need further studies to verify the biased accuracy from the double counting issue by securing a larger training size.

## 4. Conclusions

In this study, we identified candidate genes for growth- and production-related traits in purebred Korean Duroc pigs, and evaluated and compared the accuracy of genomic prediction between two genotyping platforms, response variables, and two Bayesian methods (BayesB and BayesC). A total of 15 and 12 informative 1 Mb window regions for growth- and production-related traits were identified using the Illumina60K and GeneSeek80K panels, respectively. The genomic accuracy when using DEBVincPA as the response variable was of higher value than other response variables. We suggest that a fine-mapping study is necessary to pinpoint the causal variant of the informative genomic region (i.e., the MC4R gene), and that the genomic accuracy for growth- and production-related traits will be improved by adding a pinpoint for the causal variant of the informative genomic region. Furthermore, a genomic selection model for growth- and production-related traits could be useful for future genomic evaluation in purebred Korean Duroc pigs.

## Figures and Tables

**Table 1:** Variance components and heritability estimated for growth- and production-related traits in Duroc pigs.

Trait ^1^	Additive Genetic Variance	Phenotypic Variance	Heritability
BFAT	1.21	3.42	0.35
DAYS	34.57	85.10	0.41
LMA	1.14	7.15	0.16
PCL	2.09	5.52	0.38

^1^ BFAT = backfat thickness; DAYS = days to 90 kg body weight; LMA = loin muscle area; PCL = lean percent.

**Table 2:** Comparison of relationships among animals within and across clusters in K-means 10-fold cross validations.

No. of Clusters	No. of Animals	inBreC ^1^	*a*_max_within_^2^	*a*_max_between_^3^	*a*_ij_within_^4^	*a*_ij_between_^5^
1	94	0.011	0.48 (0.11)	0.32 (0.14)	0.09 (0.02)	0.05 (0.01)
2	162	0.031	0.47 (0.12)	0.17 (0.10)	0.07 (0.01)	0.01 (0.01)
3	78	0.048	0.52 (0.10)	0.37 (0.13)	0.19 (0.03)	0.05 (0.00)
4	113	0.070	0.54 (0.11)	0.40 (0.11)	0.19 (0.03)	0.05 (0.00)
5	61	0.048	0.52 (0.11)	0.39 (0.14)	0.17 (0.03)	0.05 (0.01)
6	65	0.053	0.55 (0.08)	0.37 (0.13)	0.23 (0.02)	0.05 (0.01)
7	70	0.029	0.42 (0.15)	0.39 (0.12)	0.10 (0.03)	0.04 (0.01)
8	112	0.009	0.49 (0.10)	0.26 (0.10)	0.10 (0.03)	0.03 (0.00)
9	123	0.063	0.54 (0.10)	0.19 (0.08)	0.17 (0.02)	0.03 (0.01)
10	94	0.001	0.34 (0.19)	0.12 (0.14)	0.03 (0.02)	0.01 (0.01)

^1^ inBreC = inbreeding coefficients within clusters; ^2^ *a*_max_within_ = the average of *a*_max_ value (the maximum value of relationships for each individual) within clusters; ^3^ *a*_max_between_ = the average of *a*_max_ values between clusters (training and testing); ^4^ *a*_ij_within_ = the average of *a*_ij_ values (relationships) within clusters; ^5^ *a*_ij_between_ = the average of *a*_ij_ values between clusters (training and testing).

**Table 3:** Informative 1 Mb genome windows and significant single nucleotide polymorphisms (SNPs) based on the Bayes factor within windows associated with growth- and production-related traits in Korean Duroc pigs from the genome-wide association study (GWAS) using markers on the Illumina PorcineSNP60 genotyping platform.

Trait ^1^	SSC _Mb	GV (%) ^2^	Informative SNP	Position (Mb)	Effect	BF ^3^	Region Annotation	Gene Annotation
BFAT	1_62	1.26	MARC0038944	62.12	−0.04	24.17	intergenic	CGA (dist = 131054)
			ALGA0003581	62.20	−0.04	23.39	intergenic	CGA (dist = 44656)
			ALGA0003583	62.23	−0.04	22.52	intergenic	CGA (dist = 16224)
			ALGA0003587	62.24	0.03	21.88	intergenic	CGA (dist = 2593)
	13_205	1.24	ASGA0059825	205.31	0.12	136.38	intergenic	CLDN8 (dist = 1144428), SOD1 (dist = 309936)
	4_16	0.81	ASGA0018674	16.88	0.10	97.55	intergenic	FBXO32 (dist = 210423), DERL1 (dist = 213410)
DAYS	7_124	1.58	ASGA0093614	124.68	0.94	708.50	intergenic	BDKRB2 (dist = 26181)
	18_29	1.50	ALGA0097693	29.01	0.97	240.97	intergenic	TSPAN12 (dist = 1290900), CFTR (dist = 1388059)
	1_177	1.30	ASGA0004988	177.53	−0.66	85.63	intergenic	RNF152 (dist = 468819), MC4R (dist = 1019391)
	10_27	0.99	H3GA0029615	27.03	−0.62	77.98	intergenic	MIR181A-1 (dist = 601150), NR5A2 (dist = 252919)
	10_26	0.80	H3GA0029613	26.91	−0.58	69.77	intergenic	MIR181A-1 (dist = 489646), NR5A2 (dist = 364423)
LMA	5_87	2.36	ALGA0033240	87.39	0.21	934.40	intergenic	SLC5A8 (dist = 318494), NR1H4 (dist = 399478)
	11_68	2.02	CASI0007856	68.91	−0.18	864.04	intergenic	DCT (dist = 757772)
	1_179	1.52	ALGA0006660	179.02	0.14	57.55	intergenic	PMAIP1 (dist = 161261), MIR122 (dist = 897655)
			ALGA0006655	179.00	0.12	47.49	intergenic	PMAIP1 (dist = 144947), MIR122 (dist = 913969)
	16_9	1.40	ALGA0101487	99.10	0.09	96.92	-	NONE
	18_12	1.25	ASGA0078904	12.62	−0.05	42.71	intergenic	ZC3HAV1 (dist = 1552776), PTN (dist = 273756)
			M1GA0023069	12.64	0.05	38.94	intergenic	ZC3HAV1 (dist = 1572284), PTN (dist = 254248)
	8_128	1.24	ALGA0115575	128.24	−0.13	149.93	intergenic	NFKB1 (dist = 573086), PPP3CA (dist = 224770)
PCL	13_205	1.08	ASGA0059825	205.31	−0.18	190.47	intergenic	CLDN8 (dist = 1144428), SOD1 (dist = 309936)
	1_62	0.90	ALGA0003581	62.20	0.04	21.72	intergenic	CGA (dist = 44656)
			MARC0038944	62.12	0.04	21.00	intergenic	CGA (dist = 131054)
			ALGA003583	62.23	0.04	20.59	intergenic	CGA (dist = 16224)

^1^ BFAT = backfat thickness; DAYS = days to 90 kg body weight; LMA = loin muscle area; PCL = lean percent; ^2^ GV (%) = Percentage of additive genetic variance explained by SNP markers within each 1 Mb window region; ^3^ BF = Bayes factor.

**Table 4:** Informative 1 Mb genome windows and significant SNPs based on the Bayes factor within windows associated with growth- and production-related traits in Korean Duroc pigs from the GWAS using markers on the GeneSeek-Neogen PorcineSNP80 genotyping platform.

Trait ^1^	SSC _Mb	GV (%) ^2^	Informative SNP	Position (Mb)	Effect	BF ^3^	Region Annotation	Gene Annotation
BFAT	1_178	1.88	WU_10.2_1_178188861	178.19	−0.21	195.56	intergenic	RNF152(dist = 1123583), MC4R (dist = 364627)
	18_58	1.06	WU_10.2_18_58809866	58.81	−0.04	26.71	intergenic	INHBA (dist = 800771)
	1_62	1.04	ALGA0003581	62.20	−0.03	21.94	intergenic	CGA (dist = 44656)
			ALGA0003587	62.24	0.03	21.51	intergenic	CGA (dist = 2593)
	14_150	0.93	WU_10.2_14_150298075	150.30	0.08	68.20	intergenic	GLRX3 (dist = 891194)
			M1GA0019859	150.87	0.03	21.50	intergenic	GLRX3 (dist = 891194)
DAYS	18_29	1.19	ALGA0097693	29.01	0.78	145.57	intergenic	TSPAN12 (dist = 1290900), CFTR (dist = 1388059)
	14_4	1.16	WU_10.2_14_4968099	4.97	0.86	186.71	intergenic	LPL (dist = 511359), DOK2 (dist = 1649547)
	1_177	0.99	ASGA0004988	177.53	−0.51	58.27	intergenic	RNF152 (dist = 468819), MC4R (dist = 1019391)
LMA	1_178	3.56	WU_10.2_1_178188861	178.19	0.30	1010.87	intergenic	RNF152 (dist = 1123583), MC4R (dist = 364627)
	5_87	2.17	ALGA0033240	87.39	0.20	659.04	intergenic	SLC5A8 (dist = 318494), NR1H4 (dist = 399478)
	11_68	1.19	CASI0007856	68.91	−0.12	158.68	intergenic	DCT (dist = 757772)
	16_9	0.97	ALGA0101487	9.91	0.08	88.43	-	NONE
	8_128	0.85	ALGA0115575	128.24	−0.09	77.91	intergenic	NFKB1 (dist = 573086), PPP3CA (dist = 224770)
PCL	1_178	1.44	WU_10.2_1_178188861	178.19	0.26	167.70	intergenic	RNF152 (dist = 1123583), MC4R (dist = 364627)
	11_74	0.61	WU_10.2_11_74507674	74.51	0.12	85.22	intergenic	IPO5 (dist = 334379), SLC15A1 (dist = 382105)

^1^ BFAT = backfat thickness; DAYS = days to 90 kg body weight; LMA = loin muscle area; PCL = lean percent; ^2^ GV (%) = Percentage of additive genetic variance explained by SNP markers within each 1 Mb window region; ^3^ BF = Bayes factor.

**Table 5:** Accuracies and their standard errors of genomic prediction between molecular breeding values and their corresponding response variables (DEBVexcPA or DEBVincPA) and according to Bayesian methods and SNP genotyping platforms (Illumina PorcineSNP60 and GeneSeek-Neogen PorcineSNP80) in Duroc pigs across growth- and production-related traits.

SNP Platforms	Bayes Types	Traits ^1^	Response Variables ^2^	
			DEBVexcPA	DEBVincPA
Illumina60K	BayesB	BFAT	0.18 (0.044)	0.25 (0.043)
		DAYS	0.19 (0.046)	0.27 (0.044)
		LMA	0.23 (0.041)	0.30 (0.040)
		PCL	0.22 (0.045)	0.29 (0.043)
	BayesC	BFAT	0.18 (0.044)	0.26 (0.042)
		DAYS	0.19 (0.046)	0.28 (0.044)
		LMA	0.23 (0.041)	0.31 (0.040)
		PCL	0.22 (0.045)	0.30 (0.043)
GeneSeek80K	BayesB	BFAT	0.18 (0.044)	0.25 (0.042)
		DAYS	0.21 (0.046)	0.27 (0.044)
		LMA	0.25 (0.040)	0.33 (0.040)
		PCL	0.22 (0.045)	0.30 (0.043)
	BayesC	BFAT	0.18 (0.044)	0.25 (0.042)
		DAYS	0.20 (0.046)	0.27 (0.044)
		LMA	0.24 (0.041)	0.32 (0.040)
		PCL	0.22 (0.045)	0.30 (0.043)

^1^ BFAT = backfat thickness; DAYS = days to 90 kg body weight; LMA = loin muscle area; PCL = lean percent. ^2^ DEBVexcPA = deregressed-EBV excluding parent average; DEBVincPA = deregressed-EBV including parent average.
